# Lower Extremity Muscle Activity During a Women’s Overhand Lacrosse Shot

**DOI:** 10.2478/hukin-2014-0028

**Published:** 2014-07-08

**Authors:** Brianna M. Millard, John A. Mercer

**Affiliations:** 1 Department of Kinesiology and Nutrition Sciences, University of Nevada, Las Vegas.

**Keywords:** electromyography, sport skill, technique, biomechanics

## Abstract

The purpose of this study was to describe lower extremity muscle activity during the lacrosse shot. Participants (n=5 females, age 22±2 years, body height 162.6±15.2 cm, body mass 63.7±23.6 kg) were free from injury and had at least one year of lacrosse experience. The lead leg was instrumented with electromyography (EMG) leads to measure muscle activity of the rectus femoris (RF), biceps femoris (BF), tibialis anterior (TA), and medial gastrocnemius (GA). Participants completed five trials of a warm-up speed shot (Slow) and a game speed shot (Fast). Video analysis was used to identify the discrete events defining specific movement phases. Full-wave rectified data were averaged per muscle per phase (Crank Back Minor, Crank Back Major, Stick Acceleration, Stick Deceleration). Average EMG per muscle was analyzed using a 4 (Phase) × 2 (Speed) ANOVA. BF was greater during Fast vs. Slow for all phases (p<0.05), while TA was not influenced by either Phase or Speed (p>0.05). RF and GA were each influenced by the interaction of Phase and Speed (p<0.05) with GA being greater during Fast vs. Slow shots during all phases and RF greater during Crank Back Minor and Major as well as Stick Deceleration (p<0.05) but only tended to be greater during Stick Acceleration (p=0.076) for Fast vs. Slow. The greater muscle activity (BF, RF, GA) during Fast vs. Slow shots may have been related to a faster approach speed and/or need to create a stiff lower extremity to allow for faster upper extremity movements.

## Introduction

Participation in the sport of lacrosse has increased nationwide (Hinton et al., 2005). Although the sport has an extensive history, there is a paucity of lacrosse specific research. There is some emerging research on lacrosse injuries (Bowers et al., 2010; Dick et al., 2007; Elkousy et al., 2000; Hinton et al., 2005; Lincoln et al., 2007), conditioning/testing techniques (Gutowski and Rosene, 2011; Pistilli et al., 2008), shot accuracy (Marsh et al., 2010), passing kinematics (Livingston, 2006), shooting kinematics (Crisco et al., 2009), ball characteristics (Crisco et al., 2005), player characteristics (Schmidt et al., 1981; Shaver, 1980), and some early work on teaching lacrosse skills (Barrett and Collie, 1996).

Although lacrosse specific research is emerging, there are minimal descriptive data of the lacrosse shot. It is understood that the reason for the paucity of lacrosse specific research is partly because the sport is growing quickly and has not been adequately researched and partly because there is a wide variety of ways to shoot during a lacrosse game. For example, during a game situation, a shooter may be dodging a defender, receiving a pass and having to shoot quickly, or shooting on the run. The dynamic nature of the task makes it difficult to replicate in a laboratory setting. However, anecdotally, it is common to rank the player skill level based upon shot speed where the shooter simply approaches the net in a way to achieve maximal shot velocity. This type of shot can be replicated in the laboratory setting allowing specific measurements to be made in a way to understand the critical features of achieving a fast and accurate shot. Using this model, Mercer and Nielson (2012) provided a description of the lacrosse shot that included six phases of a shot: Approach, Crank Back Minor, Crank Back Major, Stick Acceleration, Stick Deceleration, and Follow Through/Recovery (Table 1).

Considering the movements in this model, it would seem that the lower extremity movements are critical in developing shot speed. However, there is no research on how active muscles are causing the movements during a shot. Furthermore, there are no data on females shooting a lacrosse shot. Since the shaft and head for women’s vs. men’s lacrosse, it makes sense to study each group individually.

Given the paucity of research in this area and based upon some pilot work, it was decided to investigate muscle activity of the lead leg (i.e., the forward most leg planted during the shot) for female lacrosse players shooting a lacrosse ball. Specifically, the purpose of this study was to describe lower extremity muscle activity during the lacrosse shot for women. Furthermore, the intent of this research was to compare how active select lower extremity muscles were between phases as well as between two different shot speeds.

## Material and Methods

### Participants

Participants (n=5 females, age: 21.8 ± 2 years, body height: 162.6 ± 15.2 cm, body mass: 63.7 ± 23.6 kg, years played: 7.2 ± 14 years, hand dominance: right (5), lead leg: left (5), primary position: defense (2), midfield (1), offense (2)) were free from injury and had at least one year of lacrosse experience. All participants read and signed a university approved informed consent form before participating in the study.

### Instrumentation

Muscle activity was measured using an 8-channel telemetry EMG system (TeleMyo 2400T, G2; Noraxon USA Inc., Scottsdale, AZ; 1500Hz). Duel electrodes (Ambu Blue Sensor N, Noraxon USA Inc. Scottsdale, AZ) were placed in line with the muscle fibers on the surface of the skin following manufacturer guidelines for lead placement. Video was recorded with a Panasonic Digital Video Camera Recorder (Panasonic NV-GS37, Secaucus, NJ). Speed was measured using a radar gun (Stalker Pro II, Applied Concepts, Inc. /Stalker Radar, Plano, TX) placed immediately behind the goal at approximately mid-goal height.

### Procedure

Participants were instructed to wear their own shoes appropriate for shooting in an indoor gymnasium and comfortable practice clothing. Electromyography (EMG) data were obtained by first cleaning the electrode placement sites with alcohol pads and (if necessary) shaving any hair. The rectus femoris (RF), biceps femoris (BF), medial gastrocnemius (GA), and tibialis anterior (TA) of the lead leg were instrumented. The ground lead was placed in combination with the TA. In addition, the lateral GA was instrumented but it was determined after data collection that noise was present in this muscle for two participants. Given the low number of participants, it was decided to drop this muscle from analysis and use only the medial GA. Prior to testing, participants completed a 5 s maximal voluntary isometric contraction (MVIC) for each muscle. Subjects were given instructions and practiced the MVIC prior to testing. During MVIC testing, verbal encouragement was given. The RF and BF were tested while the subject was sitting with the knee at 90° flexion and tester providing enough resistance to maintain isometric contraction. The TA was tested while sitting and the researcher was providing resistance to dorsiflexion and the GA tested while the subject stood and contracted to cause plantarflexion. The average, full-wave rectified average MVIC was used to normalize EMG data per muscle.

All participants used their own stick and shot with their dominant side. A marker was placed on the ground 3 m from the goal and participants were instructed to release the ball from this point. They were allowed to start the shot from 9 m from the goal. Participants completed two throwing conditions: a warm up speed (Slow) and a game speed (Fast). Participants completed five trials per condition with trials considered valid as long as the speed was within 2.2 m/s of the previous shot and within a 4.5 m/s range. All shots were registered by the camera set such that specific discrete events could be identified (but the camera was not set specifically to capture movements in a single plane of motion). Condition order was always Slow - Fast. For each trial, data collection began before the participant initiated the shot and continued until the completion of the Follow Through.

### Data Reduction

The video record was used to identify the discrete events defining each phase and the times the events occurred (Table 1). Since all participants shot right handed, the lead foot (front most foot at the time of shooting) was the left, drive foot the right, and the top arm (hand closest to the head of the shaft) was the right.

For the purpose of this study, the phases that were analyzed were Crank Back Minor, Crank Back Major, Stick Acceleration, and Stick Deceleration. The time of discrete events defining each of these phases was recorded. Electromyography data for each muscle were processed by removing any zero offset, full-wave rectifying the data, normalizing to MVIC, and averaging within each phase (Average EMG).

### Statistical Analysis

Average EMG was analyzed using a 4 (Phase) × 2 (Speed) ANOVA (SPSS, version 20.0) for each muscle (BF, RF, GA, TA) (*α*=0.05). If an interaction was observed for a muscle, paired t-tests were used to compare the average EMG for that muscle between Speeds for each Phase. For example, if a muscle was influenced by the interaction of Phase and Speed, paired t-tests were used to compare average EMG of that muscle between slow and fast shots for each of the four phases (i.e., 4 paired t-tests).

## Results

**Figure 1:**
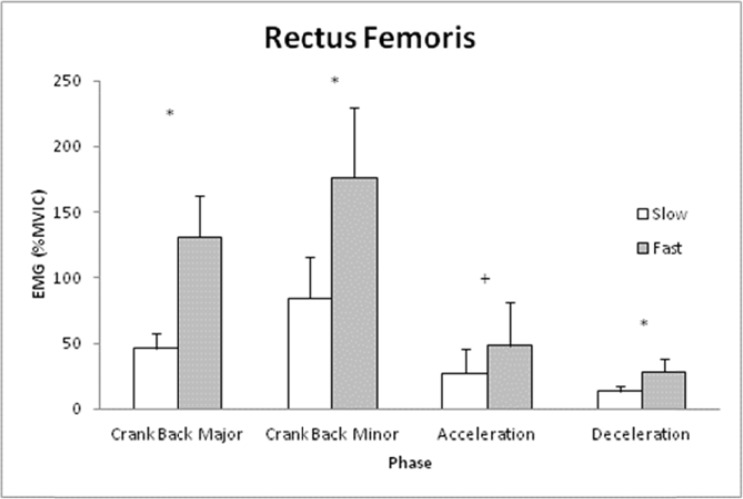
Means and standard deviations for Rectus Femoris muscle activity percent Maximal Voluntary Isometric Contraction (%MVIC)) for each phase of the lacrosse shot during slow and fast shot speeds. Note: ^*^ indicates difference between shots for that phase (p<0.05) and + indicates trend for difference (p=0.076).

Warm-up (Slow) shot speed was 15.2±4.3 m/s while game shot (Fast) speed was 19.1±4.2 m/s. The RF was influenced by the interaction of Phase and Speed (Figure 1, p<0.05). Using post hoc testing, it was determined that the RF was greater during Fast vs. Slow during Crank Back Minor and Major phases as well as Stick Deceleration phase (p<0.05), but not different between shots for Stick Acceleration (p=0.076).

**Figure 2:**
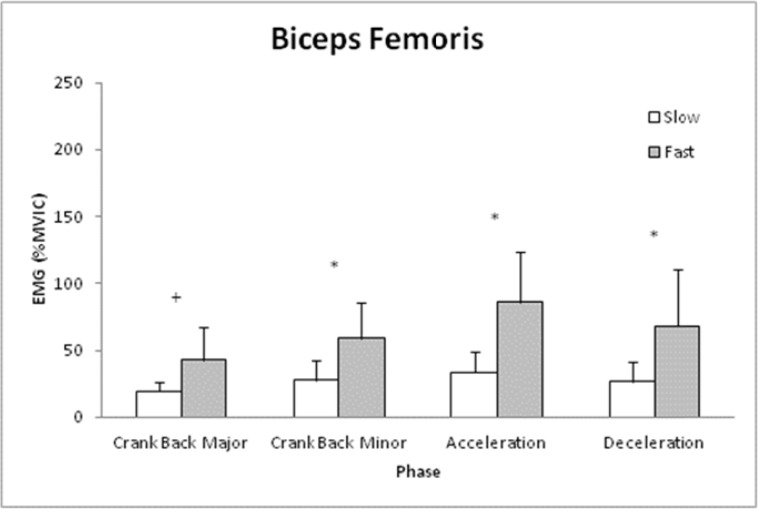
Means and standard deviations for Biceps Femoris muscle activity (percent Maximal Voluntary Isometric Contraction (%MVIC)) for each phase of the lacrosse shot during slow and fast shot speeds. Note: ^*^ indicates difference between shots for that phase (p<0.05) and + indicates trend for difference (p=0.053).

The BF was not influenced by the interaction of Phase and Speed (Figure 2, p>0.05) and was significantly different between the Phases regardless of Speed (p<0.05) but there was no main effect for Speed (p>0.05). However, since there was a trend for an interaction effect (p=0.062), post hoc tests were ran and it was determined that BF was greater during the Fast vs. Slow shot for Crank Back Major, Stick Acceleration, and Stick Deceleration phases (p<0.05). Interestingly, BF also tended to be greater during Crank Back Minor (p=0.053).

**Figure 3:**
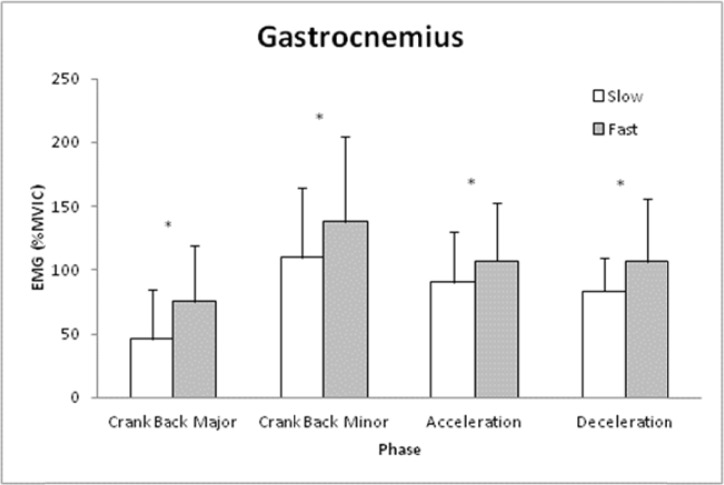
Means and standard deviations for Gastrocnemius muscle act vity (percent Maximal Voluntary Isometric Contraction (%MVIC)) for each phase of the lacrosse shot during slow and fast shot speeds. Note: ^*^ indicates difference between shots for that phase (p<0.05).

The GA was influenced by the interaction of Phase and Speed (Figure 3, p<0.05) with EMG being greater during the Fast vs. Slow shot for each Phase (p<0.05). The TA was not influenced by the interaction of Phase and Speed (p>0.05) nor was there a main effect for Phase or Speed (p>0.05).

## Discussion

This is the first study of muscle activity during women’s lacrosse shooting. More specifically, this is the first study of lower extremity muscle activity during women performing a lacrosse shot using warm up and game shot speeds. The most important observation of this work was that shot speed did not influence all muscles in the same way. We observed that BF, RF, and GA were more active during fast vs. slow shots whereas TA was not influenced by shot speed.

Although there are no other research studies on EMG of the lacrosse shot for women lacrosse players, there is research on EMG during other throwing movements. For example, Oliver et al. (2011) investigated upper and lower extremity EMG during the softball pitch. Using MG and video concurrently, it was reported that there was consistent activation of lower extremity muscles throughout phases of the pitch. Oliver et al. (2011) conjectured this was related to the need to stabilize the lower extremity to allow for upper extremity movements. In our study, we observed muscle activity that varied across phases which is in contrast to Oliver et al. (2011).

Although the phases during a softball pitch and lacrosse shot share some common elements (e.g., acceleration, deceleration), there are some unique aspects of each throwing movement. For example, during the lacrosse shot, there is an approach phase during which the player moves forward while at the same time preparing to shoot (i.e., Crank Back Major and Minor phases). In contrast, the softball pitch is performed without an approach phase due to the rules of the pitch requiring foot contact with the pitch plate. Given that the lacrosse shot allows for an approach, it seems reasonable to expect that muscle activity would vary (as observed) across phases. Another critical movement difference between the softball pitch and lacrosse shot is that the softball pitch is ‘underhand’ whereas the lacrosse shot is ‘overhand’. It may be that lower extremity muscle activity during the underhand softball pitch is different than during an overhand lacrosse shot simply because of the upper extremity movements are different.

Yamanouchi (1998) investigated lower extremity muscle activity during a baseball pitch (‘overhand’). In this study, the focus was on comparing lower extremity muscle activity of skilled and non-skilled players during pitching and it was reported that EMG of the skilled players was significantly higher than that of non-skilled group. It was partly suggested that the difference in EMG was related to pitch speed of the two groups. Similarly, we observed that BF, RF, and GA lower extremity muscle activity was influenced by lacrosse shot speed and when muscle activity was different, it was always greater during the fast vs. slow lacrosse shot. However, we did not assess the skill level of the player and future research is needed to determine the influence of skill on muscle activity during the shot.

Although a greater muscle activity during the fast vs. slow shot may be intuitive, it was interesting that an ankle dorsiflexor (TA) was not influenced by shot speed and that the knee extensor (RF), and ankle plantar flexor (GA) were each more active during the Crank Back phases for the fast vs. slow shots. Furthermore, the knee flexor (BF) tended to be greater during Crank Back Minor (p=0.053) and was more active during Crank Back Major phase. The Crank Back Minor phase begins with drive leg contact and ends with lead leg contact. In the present study, muscle activity was only recorded from the lead leg. It would be interesting to determine muscle activity of the drive leg as well in future studies. In any case, it seems that even though the lead leg was not in contact with the ground during the Crank Back Minor and even though the stick is increasing velocity in this phase, the RF, BF, and MG were more active during fast vs. slow shot speeds. This seems to be evidence for the importance of preparatory muscle activity (and/or movements) of the lower extremity in attaining faster lacrosse shot speeds. It may be that the greater activity during Crank Back Minor of these muscles is to prepare the lower extremity to be a more stable platform for shooting once lead leg contact is made. However, since the BF and RF also cause hip movements, it is not known if the difference in muscle activity when the leg is not in contact with the ground is related to hip movements. Along with that, it may be that muscle activity of the lower extremity is influenced by approach speed (which we did not quantify) and approach speed may vary between shot speeds. Future studies may be able to discern if there is a relationship between approach speed (vs. shot speed) and lower extremity muscle activity as well as a kinematic profile of the lower extremity during shooting.

The challenge with investigating muscle activity during a lacrosse shot is that each player has a unique skill level. That is, each player will have some unique aspect of the shot that makes it difficult to group participants. Even though our participants used an overhand shot technique, there was variability in the approach style used as well as trajectory of the stick during the shot. Furthermore, it may be that the discrete events developed for the men’s lacrosse shot may not fully apply to the women’s lacrosse shot. For example, the discrete event that defines the end of follow through is end of trunk rotation (Table 1, Mercer and Nielsen, 2012). Some participants had no or very little trunk rotation overall and therefore, it was not possible to quantify the follow through phase. In general, the shot speed of men’s lacrosse is faster as compared to the shot speed during women’s lacrosse shots. It is not known if that is a function of shot mechanics, muscle strength, and/or technique. It is suspected that differences in lacrosse rules between men’s and women’s lacrosse play a significant role in determining movement characteristics as well as shot speed. For example, the rules for stick head geometry and pocket depth are different for men’s and women’s lacrosse such that the ball is not cradled as securely in the head for women’s lacrosse. Qualitatively, women participants used a shooting technique that kept the stick vertical during the preparatory movements whereas Mercer and Nielsen (2012) illustrated a more horizontal stick position for men’s lacrosse during preparatory movements. Future studies could focus on the kinematics of the lacrosse shot (for either men or women) to better understand which features are important to the shot.

Our study was focused on muscle activity during a lacrosse shot and this knowledge may be helpful to the development of strength training programs as well as injury preventative and rehabilitative programs. For example, there is an abundance of data that supports that lower extremity movements and strength are contributing factors underlying mechanisms for the increased incidence of knee injuries in female vs. male athletes (e.g., Liu, 1997; Colby et al., 2000; Malinzak et al., 2000). Given that BF, RF, and GA muscles were each different between shot speeds for different phases, it makes sense to develop training programs to train these muscles to meet the demands of the shot. Furthermore, since shot speed had a differential effect on muscle activity, the player should warm up in a way to gradually increase the demand placed on key muscles.

## Summary

Continued research is needed to further understand the critical features of the lacrosse shot with the aim of improving skill while also decreasing the risk of injury through developing/refining training and rehabilitation programs. This research study was the first to quantify lower extremity muscle activity for phases during a lacrosse shot for women. The important observation made was that the BF, RF, and GA were more active when shot speed increased while the TA was not influenced by shot speed. Overall, the greater muscle activity (BF, RF, GA) as shot speed increased may have been related to a faster approach speed and/or need to create a stiff lower extremity to allow for faster upper extremity movements.

## Figures and Tables

**Table 1:** Phases and discrete events of the lacrosse shot (Mercer and Nielsen, 2012). The ‘Top Arm’ is the arm in which the hand is closest to the head of the shaft; the ‘Lead Foot’ is the foot of the front most leg planted during the shot; the ‘Drive Foot’ is the foot of the trailing leg during the shot

**Phase**	**Discrete Event**
Approach	Start of the movement
Crank Back Minor	Drive foot contact
Crank Back Major	Lead foot contact
Stick Acceleration	Maximum elbow flexion of top arm
Stick Deceleration	Ball release
Follow Through	Maximum elbow Extension of top arm
Recovery	End trunk rotation
